# Untargeted Screening Based on UHPLC-HRMS of Total Folates Produced by Lactic Acid Bacteria in Fermented Milk and During Yogurt Shelf Life

**DOI:** 10.3390/foods14071112

**Published:** 2025-03-24

**Authors:** Marianna Bozzetti, Carolina Cerri, Sara Morandi, Gabriele Rocchetti, Chiara Mussio, Federica Barbieri, Giulia Tabanelli, Daniela Bassi

**Affiliations:** 1 Department for Sustainable Food Process, Università Cattolica del Sacro Cuore, 26100 Cremona, Italy; marianna.bozzetti@unicatt.it (M.B.); carolina.cerri@unicatt.it (C.C.); sara.morandi2@unicatt.it (S.M.); chiara.mussio@unicatt.it (C.M.); 2 Department of Animal Science, Food and Nutrition, Università Cattolica del Sacro Cuore, 29122 Piacenza, Italy; gabriele.rocchetti@unicatt.it; 3 Department of Agricultural and Food Sciences, University of Bologna, 47521 Cesena, Italy; federica.barbieri16@unibo.it (F.B.); giulia.tabanelli2@unibo.it (G.T.)

**Keywords:** folate, lactic acid bacteria, biofortification, microbiological assay, UHPLC-HRMS, fermented milk

## Abstract

Folate deficiency is a widespread nutritional issue, and biofortifying dairy products through lactic acid bacteria (LAB) is a promising strategy to enhance natural folate levels. This study aimed to develop a reliable method for selecting *Streptococcus thermophilus* and *Lactobacillus delbrueckii* subsp. *bulgaricus* strains with enhanced folate production for use as functional starter cultures. Initially, a traditional microbiological assay (MA) was used to measure folate production in 36 LAB strains isolated from fermented milks. Due to MA’s limitations, an untargeted and semi-quantitative method combining ultra-high-performance liquid chromatography (UHPLC) with high-resolution mass spectrometry (HRMS) was developed for a more comprehensive folate screening. The MA showed higher folate production in *S. thermophilus* strains (309–639 µg/L) compared to *L. delbrueckii* subsp. *bulgaricus* (up to 48 µg/L). Subsequently, nine selected LAB strains were further analyzed using the UHPLC-HRMS approach, which enabled the identification and semi-quantification of six folate metabolites, namely dihydrofolate, tetrahydrofolate (THF), 10-formyl-THF, 5,10-methenyl-THF, 5,10-methylene-THF, and 5-methyl-THF. Lab-scale yogurt production using the top-performing strains, as identified through the HRMS method, demonstrated an increase in folate content over a 14-day shelf life. These findings revealed the potential of UHPLC-HRMS as a high-throughput alternative method for folates detection, offering a promising tool for screening folate-enhanced LAB strains for biofortification.

## 1. Introduction

Folate uptake in humans presents a challenge that could be addressed through consumption of biofortified foods, where these vitamins can be synthetized through fermentation by lactic acid bacteria (LAB) [1]. Folates are a group of structurally related forms of vitamin B9/11, essential for the metabolism of animals, plants, and microbial cells [2]. They have antioxidant potential and are crucial cofactors for metabolic enzymes involved in one-carbon transfer reactions. Furthermore, they play a key role in DNA replication, repair, and methylation, as well as the synthesis of nucleic acids, certain amino acids, and vitamins [3,4]. However, humans are unable to synthesize these B vitamins [4], while plants, bacteria, and fungi can synthesize folates de novo. Certain lactic acid bacteria are known to produce folate during fermentation, with tetrahydrofolate (THF) and methyl-tetrahydrofolate (MTHF) being the main forms [5,6,7]. The ability of LAB to synthesize folate varies greatly among species and is influenced by strain differences, culture conditions, and the presence of different *fol* genes (*folE*, *folQ*, *folK*, *folP*, *folA*, and *folC*), which encode for the enzymes responsible for folate synthesis [8]. LAB produce folates intracellularly for their own growth, but some of them are also released into the surrounding medium [9]. This extracellular folate can enhance the folate content in fermented foods, providing a natural source of this vitamin without adverse effects on human health [9,10,11,12]. In fact, various studies have raised concerns regarding the safety of using chemically synthesized folic acid in foods [13].

Milk is an ideal medium for fermentation due to its nutrients and proteins, which can also help stabilize folate. Optimizing fermentation conditions using high-folate-producing LAB strains is essential to increase total folate levels. However, achieving this goal requires the availability of a sensitive detection method for accurate folate quantification and differentiation. In addition, the variability in folate forms and their low levels in foods make this testing a difficult task [14,15].

The microbiological assay (MA) is the traditional and widely recognized method for folate analysis. It relies on the growth of an indicator strain, *Lactobacillus rhamnosus* ATCC 7469, with the folate content in the culture medium being directly proportional to the growth of that microorganism [14]. Growth is assessed by measuring changes in the turbidity of the solution [14]. However, this assay has certain limitations, as other factors may influence the growth of the bacterium [15].

In recent years, there has been an increased interest in chromatographic techniques for folate analysis due to their high sensitivity, excellent specificity, and significant speed, as well as the ability to differentiate between various forms of folate [15,16]. Despite these advances, no chromatographic method has yet been officially approved as suitable for food analysis [17]. Particularly, the identification of folates in dairy products using chromatographic techniques presents several bottlenecks, largely due to the chemical nature of folates and the complexity of the sample matrix. Folates are highly unstable, being sensitive to light, heat, and pH changes, and are prone to oxidation and enzymatic degradation during extraction and analysis [18]. Dairy matrices, such as milk and yogurt, further complicate identification due to the presence of proteins, lipids, and carbohydrates, which can bind folates, especially via folate-binding proteins like caseins, and co-elute during chromatography, causing interferences [19]. The diversity of folate isoforms and their interconversion during sample processing also pose challenges for accurate profiling. Also, matrix effects can alter the ionization efficiency in UHPLC-MS, leading to quantification errors. Therefore, to overcome these issues, optimized extraction methods should include antioxidants to prevent folate oxidation, protein precipitation to release bound folates, or solid-phase extraction (SPE) for sample clean-up. Additionally, the use of high-resolution mass spectrometry (HRMS) can contribute to improving sensitivity, correcting matrix effects, and ensuring accurate quantification. Therefore, further optimization is still required for their application to different food types [15,17] and also for the evaluation of biologically synthesized folate.

The aim of this study was to select LAB strains, specifically *Streptococcus thermophilus* and *Lactobacillus delbrueckii* subsp. *bulgaricus*, with enhanced folate production capacity in a dairy matrix. An initial screening of folate-producing LAB strains was performed using a microbiological assay. Selected strains were then further examined for the presence of folate biosynthesis genes and their folate production capacity through a semi-quantitative approach that combined ultra-high-performance liquid chromatography (UHPLC) with high-resolution mass spectrometry (HRMS) data analysis. The same UHPLC-HRMS approach was then used to evaluate the total folate content in a laboratory-scale yogurt production trial fermented with different blends of the best folate-producing LAB strains during a 14-day shelf life. This study represents a first preliminary investigation on the potential of UHPLC-HRMS analysis for the estimation of extracellular folates produced by LAB in dairy matrices.

## 2. Materials and Methods

### 2.1. Bacterial Strains and Growth Conditions

A total of 36 LAB strains (Table 1), previously isolated from fermented milks or dairy products, belonging to *Streptococcus thermophilus* (26) and *Lactobacillus delbrueckii* subsp. *bulgaricus* (9) species, were assayed for their capacity to produce folate. *L. delbrueckii* subsp. *bulgaricus* strains were grown in De Man, Rogosa, and Sharpe (MRS) broth (Oxoid Ltd., Basingstoke, UK) for 16–24 h at 37 °C. *S. thermophilus* strains were cultured in M17 broth (Oxoid Ltd., Basingstoke, UK) supplemented with 2% lactose, and incubated at 37 °C overnight.

For DNA extraction, LAB strains were grown on solid media. *S. thermophilus* strains were cultivated on M17 Agar (Oxoid Ltd., Basingstoke, UK) and incubated at 42 °C overnight under anaerobic conditions, while *L. delbrueckii* subsp. *bulgaricus* strains were cultured on MRS Agar (Oxoid Ltd., Basingstoke, UK) and incubated at 37 °C overnight under the same conditions.

### 2.2. Folate Quantification in Milk Based on Microbiological Assay

Cultures of *S. thermophilus* and *L. delbrueckii* subsp. *bulgaricus*, grown overnight in their respective media, were centrifuged at 4000 rpm for 15 min. The cells were washed and resuspended in an equal volume of saline solution (0.9% NaCl). Each strain was then added to skim milk (Oxoid Ltd., Basingstoke, UK) with an inoculum of 2% and incubated at 42 °C for 8 h, until coagulation occurred (pH 4.5–4.8).

An initial screening of extracellular folate production in milk was performed using the MA method [20], with minor modifications. *Lactobacillus rhamnosus* ATCC 7469, an auxotrophic strain for folate, was used as an indicator strain [21]. After overnight culture at 37 °C, cells of the indicator strain were harvested by centrifugation, washed with saline solution, inoculated (2% *v*/*v*) into double-concentrated Folic Acid Casei Medium (Difco Laboratories, Detroit, MI, USA), and loaded in triplicates into a 96-well microplate prepared as follows. An amount of 500 µL of fermented milk samples was collected and combined with 500 µL of saline solution. The samples were then mixed and boiled at 100 °C for 5 min, followed by centrifugation at 10,000 rpm for 6 min. The supernatants were collected and diluted 1:40 in a Sodium Phosphate Buffer (PBS) solution. The diluted samples were added in triplicate to the 96-well microplate. A standard curve was realized using a standard solution of folic acid (Sigma-Aldrich, St. Louis, MO, USA) diluted in a range from 0.0078 to 10 ng/mL in PBS, for a total of 12 standards. Plates were incubated at 37 °C for 24 h under anaerobic conditions. After the incubation period, the growth of the indicator strain was measured by optical density (OD) at 620 nm using a microplate reader. Total folate concentration was computed according to the linear regression of the standard curve. The final concentration of folate produced by tested strains was expressed in µg/L.

### 2.3. DNA Extraction and Molecular Identification of Folate-Producing Genes

A total of 11 LAB strains, including 9 *S. thermophilus* and 2 *L. delbrueckii* subsp. *bulgaricus*, were selected based on the results of the MA. The presence of four genes (Table 2) responsible for the production of folate was investigated in these strains. The oligonucleotides listed in Table 2 were designed to amplify *fol* genes on the basis of the comprehensive genome sequence of *L. delbrueckii* subsp. *bulgaricus* ATTC 11842.

The chromosomal DNA was extracted from pure colonies of the strains cultured overnight through microLYSIS^®^—Plus DNA (Microzone Ltd., Haywards Heath, UK), following the manufacturer’s recommended protocol.

The PCR assay was performed in a final volume of 25 µL, including Master Mix PCR 2X (Promega, Gutenbergring, Germany), 1 µM forward primer, 1µM reverse primer, and 2 µL of bacterial DNA. The reaction was conducted by applying the following thermal conditions: 5 min at 94 °C; 35 cycles of 94 °C for 30 s, 50 °C for 30 s, 72 °C for 30 s, and 72 °C for 7 min.

The amplification products were observed on a 1% agarose gel with SyBR^®^ Safe (Invitrogen, Grand Island, NY, USA). Both negative and positive controls were included in each reaction.

### 2.4. Yogurt Preparation

Three strains of *S. thermophilus* and one strain of *L. delbrueckii* subsp. *bulgaricus* (Table 3) were used as starter cultures for biofortified yogurt production. The yogurts were prepared using UHT cow’s milk from a local supermarket.

Bacterial strains were grown in milk as previously described and, for each formulation (Table 3), inoculated with a 2:1 ratio in 25 mL of UHT milk. Samples were incubated at 42 °C until reaching a pH of approximately 4.6. The yogurts were stored at a temperature of 4 °C for 14 days. Three biological replicates (*n* = 3) were made for each sample.

### 2.5. Viability of Lactic Acid Bacteria and Folate Detection During Yogurt Shelf Life

The viability of the LAB strains used for yogurt production was monitored by microbiological counts on agar plates at three different storage times: initial time point (T0), after 7 days (T7), and after 14 days (T14) during the shelf life. For each sample, serial dilutions were prepared and spread on MRS and M17 Agar supplemented with lactose (2% *v*/*v*) for the counts of *L. delbrueckii* subsp. *bulgaricus* and *S. thermophilus*, respectively [22]. MRS and M17 plates were incubated anaerobically for 48 h at 37 °C and 42 °C, respectively.

To enable a comparative analysis of the experimental samples with a commercially available product, a yogurt containing a combination of *L. delbrueckii* subsp. *bulgaricus*, *S. thermophilus*, *Bifidobacterium* spp., and *L. casei* was purchased from a local company. Microbiological counts for *L. delbrueckii* subsp. *bulgaricus* and *S. thermophilus* were also performed for the commercial yogurt at T0, T7, and 14, as described above.

The quantification of total folate metabolites in the experimental and commercial yogurt samples was performed at T0, T7, and 14 using the method described in Section 2.6.

### 2.6. Extraction and Semi-Quantitative Analysis of Folates Based on UHPLC-HRMS in Milk and Yogurt Samples

In this work, we optimized a novel extraction method for total folates in dairy matrices. A total of 200 µL of each milk and yogurt sample was combined into a 2 mL Eppendorf tube with 400 µL of an extracting solution containing 80% methanol and 3% formic acid (*v*/*v*). The mixture was subjected to ultrasound-assisted extraction (UAE; DU-32 ARGOLab, Milan, Italy), working at 25 °C, for 10 min at 120 W. Afterwards, the extracted samples were centrifuged at 14,000 rpm, at 4 °C for 15 min, and then incubated overnight at −18 °C, to promote protein precipitation. Thereafter, supernatants were filtered through 0.22 µm syringe filters and transferred into UHPLC vials until further instrumental analysis.

Estimation of the extracellular total folates was performed through a semi-quantitative approach based on ultra-high-performance liquid chromatography (UHPLC) combined with high-resolution mass spectrometry (HRMS) data analysis. The semi-quantification of folates was performed in both the skim milk and yogurt samples. A Q-Exactive™Focus Hybrid Quadrupole-Orbitrap Mass Spectrometer (Thermo Scientific, Waltham, MA, USA) equipped with a heated electrospray ionization (HESI) probe was used. The mobile phase consisted of water acidified with 0.1% (*v*/*v*) formic acid (phase A) and methanol acidified with 0.1% (*v*/*v*) formic acid (phase B). An Agilent Zorbax Eclipse Plus C18 column (50 × 2.1 mm internal diameter, 1.8 μm particle size) (Agilent Technologies, Santa Clara, CA, USA) was used. A total run of 20 min was considered, at a constant flow rate of 0.2 mL/min. The gradient elution program was as follows: time 2 min, 90/10 A/B; time 8 min, 50/50 A/B; time 8.1 min, 5/95 A/B; time 8.5 min, 5/95 A/B; time 8.6 min, 90/10. The mass spectrometer worked in positive polarity (HESI+) and SCAN mode, with a range from 150 to 600 *m*/*z*, which corresponds to the molecular weight of folates targeted in this work, working with a nominal resolution of 70,000 FWHM at 200 m/z. The HESI source parameters were as follows: sheath gas flow rate of 40, auxiliary gas flow rate of 20, temperature of HESI probe equal to 320 °C.

The raw data from UHPLC-HRMS acquisition were then processed using MS-DIAL software (version 4.90) for post-acquisition and data filtering procedures. The process consisted of an automatic peak finding, LOWESS normalization, and annotation via spectral matching against the publicly available comprehensive database FooDB. Mass features were searched within the 150–600 *m*/*z* range, with a minimum peak height threshold of 10,000 cps. The accurate mass tolerance for peak centroiding was set at 0.05 Da for MS and 0.1 Da for MS/MS analysis. Retention time information was excluded from the total identification score calculation. The identification process relied on mass accuracy, isotopic patterns (including isotopic distribution, spacing, and abundance), and spectral matching, resulting in a total identification score with a minimum cut-off of 50%, considering the most common HESI+ adducts [23]. Additionally, the cumulative semi-quantification of extracellular total folates, including dihydrofolate, tetrahydrofolate (THF), 10-formyl-THF, 5,10-methenyl-THF, 5,10-methylene-THF, and 5-methyl-THF, in the milk and yogurt samples was performed using 5-methyl-tetrahydrofolic acid (Sigma-Aldrich, St. Louis, MO, USA) as a representative standard compound. The solution was prepared by dissolving 5 mg of 5-MTHF powder in a buffer solution composed of 1 g/L aqueous ascorbic acid, to preserve the compound from oxidation. A calibration curve (25–500 µg/L) was then built, considering a coefficient of determination > 97% (Table S1). The total folate content was expressed as µg 5-methyl-THF Equivalents (Eq.)/L (Sigma-Aldrich, Darmstadt, Germany), considering two biological replicates for each sample. Under our untargeted and HRMS semi-quantitative conditions, the matrix effect and recovery rate were evaluated on the compound 5-methyl-THF, according to the method previously reported by Matuszewski et al. [24], and considering three sets of samples, namely a standard solution sample (250 µg/L of 5-Methyl-THF in 1 g/L aqueous ascorbic acid), a fortified extracted of milk sample with 250 µg/L of 5-Methyl-THF (post-extraction spiked sample), and a fortified milk sample with 250 µg/L of 5-Methyl-THF before extraction (pre-extraction spiked sample). These preliminary trials revealed the optimum recovery rate and no significant matrix effect (Table S1). Finally, as also reported in a previous work [25], we considered the limit of quantification (LOQ) for the semi-quantitative analysis as the lowest calibration level used (25 µg/L).

### 2.7. Statistical Analysis

Extracellular folate levels obtained from the microbiological assay and yogurt microbial counts are reported as mean ± standard deviation (*n* = 3 biological replicates). Statistical analyses were performed using R software (v4.3.3, R Foundation for Statistical Computing, Vienna, Austria); a one-way analysis of variance (ANOVA) was employed, followed by Tukey’s post hoc test, with significance established at *p* < 0.05. In addition, the quantification of total folate metabolites by UHPLC-HRMS is expressed as mean ± standard deviation (*n* = 2 biological replicates), and the statistical significance was determined by ANOVA using SPSS (v26.0, IBM, Armonk, NY, USA), followed by Duncan’s post hoc test (*p* < 0.05). An unsupervised hierarchical clustering analysis was performed using MetaboAnalyst 6.0 to evaluate the accumulation patterns of various folate metabolites in milk produced by 9 selected *S. thermophilus* and 2 *L. delbrueckii* subsp. *bulgaricus* strains.

## 3. Results

### 3.1. Folate Production by Microbiological Assay and LAB Strains Selection

**Figure 1:**
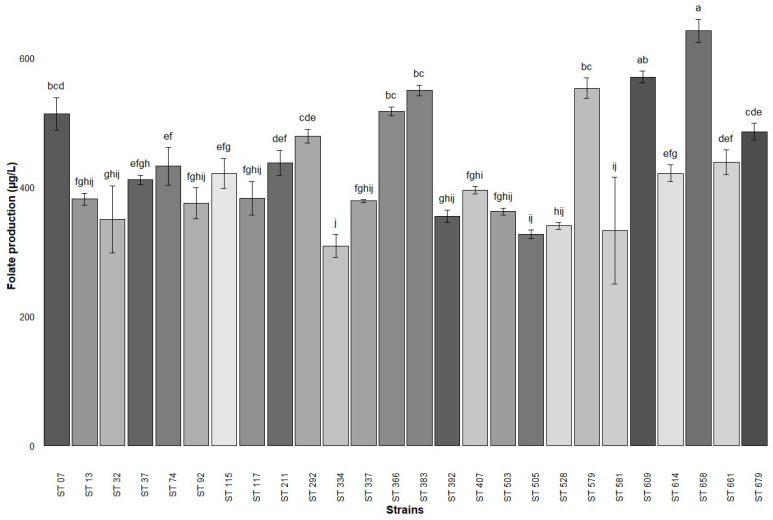
Folate production (mean ± standard deviation) by *S. thermophilus* strains in milk based on microbiological assay after a 24 h fermentation at 42 °C. The results are expressed as mean value (*n* = 3 biological replicates) ± standard deviation. Different superscript letters (^a–j^) denote significant differences between strains, as determined by one-way ANOVA coupled with a Tukey’s post hoc test (*p* < 0.05).

**Figure 2:**
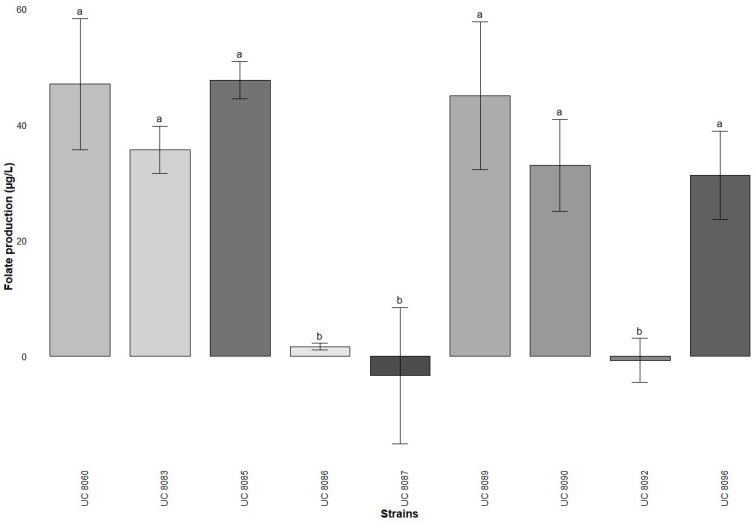
Folate production (mean ± standard deviation) by *L. delbrueckii* subsp. *bulgaricus* strains in milk based on microbiological assay after a 24 h fermentation at 37 °C. The results are expressed as mean value (*n* = 3 biological replicates) ± standard deviation. Different superscript letters (^a,b^) denote significant differences between strains, as determined by one-way ANOVA coupled with a Tukey’s post hoc test (*p* < 0.05).

An initial screening was conducted to assess the ability to produce folate of 26 *S. thermophilus* strains and 7 *L. delbrueckii* subsp. *bulgaricus* strains in milk using an MA. All *S. thermophilus* strains were found to be folate producers, with values ranging from 309 µg/L to 639 µg/L (Figure 1). From this first analysis, a total of nine strains with varying folate production levels were selected for further testing. In contrast, the *L. delbrueckii* subsp. *bulgaricus* strains exhibited significantly lower folate production, with the highest recorded value being 48 µg/L (Figure 2). Notably, UC 8087 and UC 8092 were non-productive, while UC 8086 produced only 2 µg/L. Among the remaining six *L. delbrueckii* subsp. *bulgaricus* strains that demonstrated a discrete folate production, UC 8085 and UC 8089 were selected as the two most productive strains.

### 3.2. Identification of Folate-Producing Genes

The microbiological assay allowed the selection of 11 strains (9 *S. thermophilus* and 2 *L. delbrueckii* subsp. *bulgaricus*) on the 35 tested bacteria. In order to support the results obtained, PCR analysis was performed for the identification of folate-producing genes (*folA*, *folC*, *folK*, *folP*) in the 11 selected strains. The results demonstrated that all the tested strains owned the four genes involved in the biosynthesis of folate, supporting the nature to harbor the complete genetic pathway.

### 3.3. Semi-Quantification of Folate Metabolites in Milk Through a UHPLC-HRMS Approach

A subsequent screening was performed to assess the ability of the selected nine *S. thermophilus* and two *L. delbrueckii* subsp. *bulgaricus* strains to produce folates. This was achieved by using a UHPLC-HRMS technique, which involved an untargeted metabolomics approach based on a semi-quantitative analysis of the main folate metabolites. Particularly, six folate metabolites, mainly folate precursors and products, were identified and semi-quantified as total extracellular metabolites (Table S1). The metabolites considered were dihydrofolate, THF, 10-formyl-THF, 5,10-methenylTHF, 5,10-methylene-THF, and 5-methyl-THF. The heat map reported in Table S1 identified two main clusters according to the ability to produce different folate metabolites; the first cluster consisted of strains ST 658, ST 679, ST 07, and ST 292, with the strain ST 658 possessing the most exclusive profile. On the other side, the second cluster was further divided into three sub-clusters; the first sub-cluster consisted of the 2 *L. delbrueckii* subsp. *bulgaricus* strains (UC 8085 and UC 8089), while the remaining two sub-clusters included the other *S. thermophilus* strains, thus confirming a strain-specific effect on the production of folate metabolites in milk. As a general consideration, ST 658 showed an up-accumulation of folic acid and THF, while UC 8085 was highly abundant in dihydrofolate (Table S1). Accordingly, the total folate production analyzed in skim milk stored at 42 °C for 24 h (Table 4) revealed that among the *S. thermophilus* strains tested, the ST 658 was found as the best producer, with 227.99 µg/L, followed by ST 07 (221.02 µg/L) and ST 292 (140.83 µg/L). The lowest producers were identified as ST 581, ST 407, and ST 609. The difference in folate production between the worst producer (ST 581) and the best producer (ST 658) was 169.85 µg/L folate. For what concerns the two *L. delbrueckii* subsp. *bulgaricus* strains, both exhibited good productivity, with values, respectively, of 100.12 µg/L for UC 8089 and 398.57 µg/L for UC 8085. However, from a statistical standpoint, one-way ANOVA coupled with Duncan’s post hoc test (*p* < 0.05) revealed that UC8085 produced the highest folate content (Table 4).

### 3.4. Shelf Life Study on Yogurt

**Figure 3:**
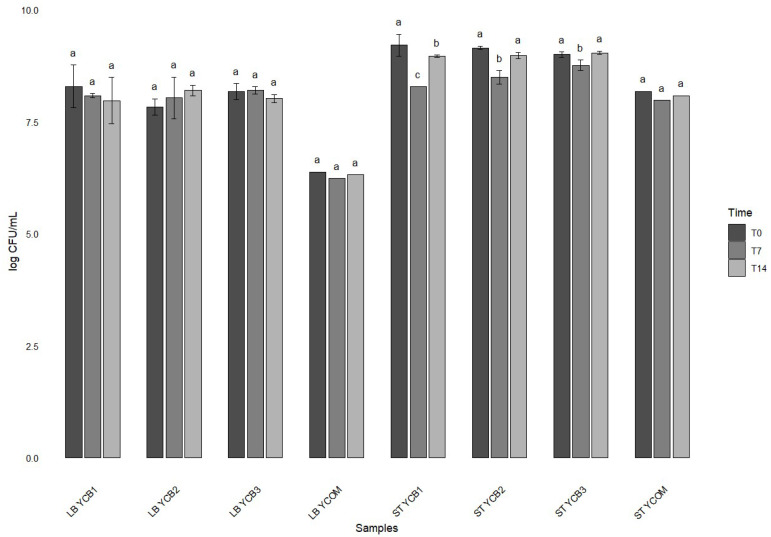
Concentration (mean log CFU/mL ± standard deviation) of *L. delbrueckii* subsp. *bulgaricus* (LB) and *S. thermophilus* (ST) in experimental (YCB1, YCB2, YCB3) and commercial (YCOM) yogurt samples during storage at 4 °C after 0 (T0), 7 (T7), and 14 (T14) days. The results are expressed as mean value (*n* = 3 biological replicates) ± standard deviation. Different superscript letters (^a–c^) denote significant differences between strains, as determined by one-way ANOVA coupled with a Tukey’s post hoc test (*p* < 0.05).

The results obtained through the untargeted UHPLC-HRMS allowed defining the best candidate strains to be used as starter cultures for yogurt production. *S. thermophilus* ST 658, ST 292, and ST 07 strains exhibited the most favorable results with regard to the total extracellular folate metabolites, while UC8085 demonstrated the best performance among the *L. delbrueckii* subsp. *bulgaricus* strains. The three *S. thermophilus* strains were therefore selected for the formulation of three blend combinations to be inoculated into cow milk, together with *L. delbrueckii* subsp. *bulgaricus* UC 8085. The initial inoculum of the selected bacterial strains was 8 log CFU/mL. The fermentation process for the production of yogurt took a total of 8 h when a final pH range between 4.68 and 4.58 was reached. The growth of both LAB was monitored throughout the 14-day shelf life and the microbiological counts are presented in Figure 3.

After fermentation, it was observed that the concentration of *S. thermophilus* strains was greater than *L. delbrueckii* subsp. *bulgaricus* UC 8085 in all three mixtures. Following a 14-day storage period, the mixture containing ST 658 and UC 8085 (YCB3) exhibited the highest concentration of bacteria. The values for *Lactobacillus* spp. were 8.20 log CFU/mL, while those for *Streptococcus* spp. were 8.76 log CFU/mL. Cumulative extracellular folates levels were then assessed in the yogurt samples with UHPLC-HRMS at the same time points, namely T0, T7, and T14 days (Table 5).

For both YCB1 and YCB2, the production of total folates significantly increased at each time point (0, 7, and 14 days). Duncan’s post hoc analysis confirmed that day 14 consistently exhibited the highest folate concentrations, significantly surpassing both days 0 and 7 (Table 5). At the end of the estimated shelf life, the mixture with the highest level was YCB1, with 432.08 µg/L. In contrast, the YCB3 mixture followed a different trend. Significant differences were observed between days 0 and 7, as well as between days 0 and 14; however, no significant difference was found between days 7 and 14. This suggests that the folate production in mix YCB3 leveled off after 7 days, with no further significant increase in folate content between 7 and 14 days. Overall, the data indicated that the folate metabolites content in the yogurt samples after 7 and 14 days was significantly greater than in the skim milk. The combination of the two bacterial strains resulted in a higher yield of folate than when the strains were cultivated independently.

The data, presented in Table 5, are also expressed as a hypothetical intake of 125 mL of yogurt, the typical daily serving size. This provides an estimate of the folate intake that could result from consuming a single serving. The highest folate intake, 54.01 µg/125 mL, corresponds to yogurt produced with the YCB1 mixture at the end of its shelf life.

Microbiological counts were also conducted on a commercial yogurt produced using a combination of *L. delbrueckii* subsp. *bulgaricus*, *S. thermophilus*, *Bifidobacterium* spp., and *L. casei*. The commercial yogurt exhibited a comparable growth profile for *Streptococcus* spp. to that observed in the experimental samples, whereas a different trend was evident for *Lactobacillus* spp. Notably, the concentration of *L. delbrueckii* subsp. *bulgaricus* remained approximately 6 log CFU/mL throughout the test period (Figure 3).

**Figure 4:**
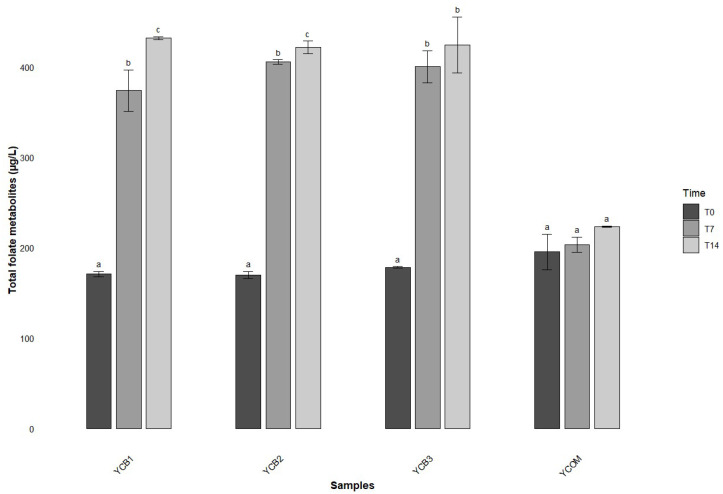
Concentration of total extracellular folate metabolites (µg/L) over the 14-day shelf life in the experimental yogurts (YCB1, YCB2, YCB3) and in commercial yogurt (YCOM). Superscript letters (^a–c^) denote significant differences between time points within each bacterial mix, as determined by Duncan’s post hoc test.

The cumulative concentration of the six folate metabolites in the commercially available yogurt was measured using UHPLC-HRMS analysis (Table 5). Similar to the experimental samples, the results from the one-way ANOVA showed a significant overall increase in folate concentrations during the storage period. However, Duncan’s post hoc test revealed no significant differences between the individual time points, indicating that the increase was relatively modest. In general, the total folate levels in the commercially available yogurt were lower than those found in the yogurts produced with the three experimental strain blends selected in the study (Figure 4).

## 4. Discussion

The present study confirmed that certain strains of *S. thermophilus* and *L. delbrueckii* subsp. *bulgaricus*, which are common starter cultures for yogurt production, are capable of biosynthesizing folate. While *S. thermophilus* is widely recognized for this ability [26], *L. delbrueckii* subsp. *bulgaricus* is generally considered a folate consumer [27], although some strains have been shown to produce this vitamin [10]. Additionally, it has been demonstrated that cells grown at low pH produce higher amounts of extracellular folate derivatives than those grown at high pH, and that *S. thermophilus* produces significant quantities of extracellular folate during growth in milk, thereby enhancing its bioavailability [4,28]. Overall, folate production is strain-dependent and influenced by culture conditions [9,29,30]. Therefore, the development of a reliable and accurate screening method for folate production in various LAB strains is crucial for their potential use in the biofortification of food products.

The conventional method for assessing folate content in food is the microbiological assay, which depends on the turbidimetric growth of *L. rhamnosus* ATCC 7469 [20] but there is an increasing need to develop more specific methods of detection. In this study, a total of 36 strains comprising *S. thermophilus* and *L. delbrueckii* subsp. *bulgaricus* strains were first screened for folate production using the standard MA. According to this method, all tested *S. thermophilus* strains demonstrated the ability to produce extracellular folates, with yields ranging from 309 to 639 µg/L, superior values if compared to those reported in previous studies [4,5,10,28]. In contrast, *Lactobacillus* strains produced lower levels of folate, two of them being non-producers, and the maximum obtained quantity reaching 48 µg/L of folate. Similar results were found by Laiño et al. [10], who tested 41 strains of *L. bulgaricus* subsp. *lactis* and found that only 4 were folate producers. This hypothesis was disproved by a recent study conducted by Hosseini et al. [4], which demonstrated that *L. delbrueckii* subsp. *bulgaricus* can produce extracellular folate in higher quantities than *S. thermophilus*, with levels exceeding 100 μg/mL. This confirms that folate production seems to be a strain-specific characteristic [12] that requests further research to be better assessed. Regarding the method, the microbiological approach, used until now in a majority of studies, presents several challenges, including interference from non-folate substances that can either stimulate or inhibit *L. rhamnosus* growth, such as variability in the incubation time and temperature, and difficulty in distinguishing between different folate derivatives [14,15,17]. These limitations have led to a growing preference for chromatographic techniques, which offer enhanced sensitivity, selectivity, and specificity [4,14,31]. Additionally, previous studies reported high repeatability and reproducibility [18] applying the HPLC approach coupled with mass spectrometry, which also allows for the differentiation and quantification of individual folate vitamers [32].

Based on this evidence, in this work, we aimed to exploit an untargeted UHPLC-HRMS method that could be a good substitute to the MA for a preliminary measurement of folate levels in dairy matrices. Under our experimental conditions, all the 11 LAB strains previously selected for the capacity to produce extracellular folates through the MA and harboring four key folate-related genes (*folA*, *folC*, *folK*, *folP*) [33] were subjected to UHPLC-HRMS methodology to confirm their classification as total folate producers and to evaluate the efficacy of this high-resolution technique. This technology permitted the semi-quantification of six folate metabolites, including dihydrofolate, THF, 10-formyl-THF, 5,10-methenyl-THF, 5,10-methylene-THF, and 5-methyl-THF (Table S1). Following this approach, the *S. thermophilus* strains showed lower folate levels than those determined by MA. This discrepancy can be attributed to the complex sample extraction and purification procedures, which may lead to the loss of sensitive folate compounds [14]. In contrast, *L. delbrueckii* subsp. *bulgaricus* demonstrated higher total folate concentrations when measured by MA. Overall, the comparison of the two techniques resulted in being quite incongruent, as the MA evaluates the growth capacity of an auxotrophic microorganism for folate, whereas UHPLC-HRMS allows for the accurate differentiation of folate vitamers. Therefore, although some improvements based on the application of a target approach together with a deconjugation step appear mandatory, the chromatography coupled with mass spectrometry method proved to be more effective in accurately determining the exact amount of total folates in the analyzed samples. However, the investigation of intracellular and extracellular folate levels should be better optimized in future research studies. Based on these considerations, and since it is reported that combining high-folate-producing strains from different LAB species may be more effective in enhancing folate content than using a single culture [1,13], we selected three *S. thermophilus* strains and one *L. delbrueckii* subsp. *bulgaricus*, according to the results of the UHPLC-HRMS analysis, to produce the experimental yogurts. The analysis revealed that all the yogurt samples exhibited an increase in total folate amount during their shelf life period. The YCB1 and YCB2 yogurts demonstrated a significant increase in total folate content after 7 and 14 days, suggesting that the LAB strain parts of these blends were capable of synthesizing or converting folate precursors into bioactive forms during both fermentation and storage. In contrast, the YCB3 formulation exhibited a significant increase in folate after 7 days, followed by a less appreciable increase at 14 days. Additionally, the commercial yogurt analyzed in this study showed consistently lower total folate concentrations compared to the experimental formulations, with no significant changes during the 14-days shelf life time. Among the tested formulations, YCB1, composed of *S. thermophilus* ST07 and *L. delbrueckii* subsp. *bulgaricus* UC 8085, emerged as the most promising, achieving a total folate concentration of 432.08 µg/L at the end of storage. Within the *L. bulgaricus* strains, UC 8085 exhibited the highest extracellular folate production, as determined by both MA and UHPLC-HRMS. Conversely, although *S. thermophilus* ST07 was not the most efficient folate producer among the tested *S. thermophilus* strains, it nonetheless demonstrated substantial folate synthesis, with concentrations of 514 µg/L and 211.02 µg/L, as measured by MA and UHPLC-HRMS, respectively. These findings confirm that the interaction between different strains may create favorable conditions for enhanced folate biosynthesis.

According to FAO/WHO (2002) [34], the Recommended Nutrient Intake (RNI) for folate is 400 µg/day for adults, 600 µg/day for pregnant women, and 200 µg/day for children aged 4–6 years. In this study, the biofortified yogurts were evaluated based on a 125 mL serving, which represents the daily portion typically consumed. Considering the bioavailable extracellular folates, none of the yogurts met the full daily folate requirements for any of the population groups. However, a food providing 10–20% of the RNI is considered a good source of folate [22,35]. The YCB1 yogurt demonstrated potential as a folate source, contributing 13.5% of the adult RNI, and 27% for children aged 4–6 years at the end of its 14-days shelf life. For both adults and children, this yogurt can be considered a good source of folate, offering an improvement over the findings of Laiño et al. [31], where their yogurt provided 10% of the RNI for adults and 20% for children. Therefore, all our experimental culture blends were demonstrated to be synergic in increasing the intake of folates in dairy food after 14 days, even shorter with respect to a standard 28-days yogurt shelf life.

## 5. Conclusions

Biofortified foods, such as yogurt, offer a promising strategy for combining micronutrient fortification with the widespread popularity of dairy products. This study explores the potential of LAB cultures for producing yogurt naturally enriched with folate. The MA traditionally employed for folate analysis has exhibited some limitations. While MA is a traditional, cost-effective method that semi-quantifies bioavailable folate forms based on the growth response of *Lactobacillus* strains to mono-, di-, and tri-glutamate forms, it remains relatively imprecise and lacks the comprehensive profiling offered by modern analytical techniques. In contrast, UHPLC-HRMS provides a more accurate, sensitive, and detailed characterization of folate species, allowing not only for precise quantification but also for the identification of the various folate isoforms. In this study, an untargeted and semi-quantitative screening using UHPLC-HRMS was also employed to identify the predominant folate species produced, providing a broader and more detailed view of the folate profile compared to MA. Although HRMS has proven effective for selecting strains and assessing total folate levels in yogurt, several challenges still remain. Developing and validating a targeted UHPLC-MS/MS technique would improve the sensitivity, specificity, and efficiency of folate analysis in dairy matrices. Optimizing extraction methods from the yogurt matrix is essential for the efficient recovery of folate and its metabolites, as well as for the quantification of both extracellular and intracellular folates. While the positive results with our bacterial blends indicate the potential of biofortified yogurt, further research is needed to explore additional LAB strains and determine the optimal folate yield during an extended yogurt shelf life. Addressing these challenges will strengthen the role of biofortified yogurt in combating folate deficiency and improving public health outcomes.

## Figures and Tables

**Table 1:** List of bacterial strains investigated in this study.

	Bacterial Strain	ID	Isolation Source
1	*Lactobacillus delbrueckii* subsp. *bulgaricus*	UC 8060	Matsoni, Georgia
2	*Lactobacillus delbrueckii* subsp. *bulgaricus*	UC 8083	Ayran, Turkey
3	*Lactobacillus delbrueckii* subsp. *bulgaricus*	UC 8085	Ayran, Turkey
4	*Lactobacillus delbrueckii* subsp. *bulgaricus*	UC 8086	Ayran, Turkey
5	*Lactobacillus delbrueckii* subsp. *bulgaricus*	UC 8087	Ayran, Turkey
6	*Lactobacillus delbrueckii* subsp. *bulgaricus*	UC 8089	Ayran, Turkey
7	*Lactobacillus delbrueckii* subsp. *bulgaricus*	UC 8090	Ayran, Turkey
8	*Lactobacillus delbrueckii* subsp. *bulgaricus*	UC 8092	Ayran, Turkey
9	*Lactobacillus delbrueckii* subsp. *bulgaricus*	UC 8096	Ayran, Turkey
10	*Streptococcus thermophilus*	ST 07	Hard cheese, Italy
11	*Streptococcus thermophilus*	ST 13	Hard cheese, Italy
12	*Streptococcus thermophilus*	ST 32	Fermented milk
13	*Streptococcus thermophilus*	ST 37	Fermented milk
14	*Streptococcus thermophilus*	ST 74	Fermented milk
15	*Streptococcus thermophilus*	ST 92	Fermented milk
16	*Streptococcus thermophilus*	ST 115	Fermented milk
17	*Streptococcus thermophilus*	ST 117	Fermented milk
18	*Streptococcus thermophilus*	ST 211	Fermented milk
19	*Streptococcus thermophilus*	ST 292	Ayran, Turkey
20	*Streptococcus thermophilus*	ST 298	Fermented milk
21	*Streptococcus thermophilus*	ST 334	Fermented milk
22	*Streptococcus thermophilus*	ST 337	Fermented milk
23	*Streptococcus thermophilus*	ST 366	Ayran, Turkey
24	*Streptococcus thermophilus*	ST 383	Ayran, Turkey
25	*Streptococcus thermophilus*	ST 392	Fermented milk
26	*Streptococcus thermophilus*	ST 407	Ayran, Turkey
27	*Streptococcus thermophilus*	ST 503	Hard cheese, Italy
28	*Streptococcus thermophilus*	ST 505	Fermented milk
29	*Streptococcus thermophilus*	ST 528	Fermented milk
30	*Streptococcus thermophilus*	ST 579	Fermented milk
31	*Streptococcus thermophilus*	ST 581	Ayran, Turkey
32	*Streptococcus thermophilus*	ST 609	Matsoni, Georgia
33	*Streptococcus thermophilus*	ST 614	Fermented milk
34	*Streptococcus thermophilus*	ST 658	Matsoni, Georgia
35	*Streptococcus thermophilus*	ST 661	Fermented milk
36	*Streptococcus thermophilus*	ST 679	Ayran, Turkey

**Table 2:** List of primers used in PCR test for identification of folate genes.

Genes	Primers	Sequence (5′ → 3′)	Size (bp)
*fol A*	folAf	AGCTACGTTTGGGCAGAAGA	489 bp
	folAr	CGGTGGGCTTCACTCTTTAC	
*fol C*	folCf	GTATTTTGCCGAACAGCGGG	1338 bp
	folCr	TCAACAAATGCGCTGATGCC	
*fol K*	folKf	GTATTTTGCCGAACAGCGGG	1074 bp
	folKr	GAAAGTTCGCGCTGCTGATT	
*fol P*	folPf	ACATTTAGCGGCAACGTCAC	1101 bp
	folPr	CTTTTTCAAGCCCAACGCCT	

**Table 3:** Yogurt LAB blends and production details.

Name of the Blend	*S. thermophilus*	*L. delbrueckii* subsp. *bulgaricus*	Ratio	% Inoculated
YCB1	ST 07	UC 8085	2:1	8:4%
YCB2	ST 292	UC 8085	2:1	8:4%
YCB3	ST 658	UC 8085	2:1	8:4%

**Table 4:** Total folate metabolites produced by *S. thermophilus* and *L. delbrueckii* subsp. *bulgaricus* strains in milk analyzed by UHPLC-HRMS. The results are expressed as mean value (*n* = 2 biological replicates) ± standard deviation. Different superscript letters (^a–e^) in the column denote significant differences between strains, as determined by one-way ANOVA coupled with a Duncan’s post hoc test (*p* < 0.05).

Strain	Total Folate Metabolites (µg/L)
ST 07	211.02 ± 4.20 ^d^
ST 292	140.83 ± 28.10 ^c^
ST 366	128.82 ± 1.90 ^bc^
ST 407	71.60 ± 12.30 ^a^
ST 503	132.68 ± 3.20 ^bc^
ST 581	58.14 ± 5.04 ^a^
ST 609	72.34 ± 2.50 ^a^
ST 658	227.99 ± 9.30 ^d^
ST 679	89.02 ± 9.50 ^ab^
UC 8085	398.57 ± 51.5 ^e^
UC 8089	100.12 ± 1.5 ^abc^

**Table 5:** Production of total extracellular folate metabolites (µg/L) in experimental and commercial yogurt samples at 0, 7, and 14 days, and estimated intake per 125 mL serving. Values are presented as mean ± standard deviation (*n* = 2). Superscript letters (^a–c^) denote significant differences between time points within each bacterial mix, as determined by Duncan’s post hoc test.

Blend	Time (Days)	Total Folate Metabolites (µg/L)	Folate Intake (µg/125 mL)
YCB1	0	170.68 ± 2.79 ^a^	21.34 ^a^
	7	373.83 ± 23.13 ^b^	46.73 ^b^
	14	432.08 ± 1.63 ^c^	54.01 ^c^
YCB2	0	169.53 ± 3.96 ^a^	21.19 ^a^
	7	405.70 ± 2.46 ^b^	50.71 ^b^
	14	421.74 ± 6.96 ^c^	52.72 ^c^
YCB3	0	178.23 ± 0.78 ^a^	22.28 ^a^
	7	400.30 ± 17.96 ^b^	50.04 ^b^
	14	424.41 ± 30.97 ^b^	53.05 ^b^
YCOM	0	195.35 ± 16.69 ^a^	24.42 ^a^
	7	203.31 ± 8.5 ^a^	25.41 ^a^
	14	223.23 ± 0.62 ^a^	27.90 ^a^

## Data Availability

The original contributions presented in the study are included in the article/Supplementary Materials, further inquiries can be directed to the corresponding author.

## Supplementary Materials

The following supporting information can be downloaded at: https://www.mdpi.com/article/10.3390/foods14071112/s1, Table S1: a supplementary Excel file containing the following sheets: (a) matrix effect and recovery trials; (b) raw annotations of folates based on UHPLC-HRMS approach; (c) hierarchical clustering heat map built considering the relative abundance values of each folate metabolites detected in the milk samples.
